# p38 MAPK-Mediated Bmi-1 Down-Regulation and Defective Proliferation in ATM-Deficient Neural Stem Cells Can Be Restored by Akt Activation

**DOI:** 10.1371/journal.pone.0016615

**Published:** 2011-01-28

**Authors:** Jeesun Kim, Jeon Hwangbo, Paul K. Y. Wong

**Affiliations:** 1 Department of Molecular Carcinogenesis, MD Anderson Cancer Center, The University of Texas, Smithville, Texas, United States of America; 2 Department of Genetic Engineering and Graduate School of Biotechnology, Kyung Hee University, Suwon, Korea; Tokyo Medical and Dental University, Japan

## Abstract

A-T (ataxia telangiectasia) is a genetic disease caused by a mutation in the *Atm* (A-T mutated) gene that leads to neurodegeneration. Despite an increase in the numbers of studies in this area in recent years, the mechanisms underlying neurodegeneration in human A-T are still poorly understood. Previous studies demonstrated that neural stem cells (NSCs) isolated from the subventricular zone (SVZ) of *Atm* ^-/-^ mouse brains show defective self-renewal and proliferation, which is accompanied by activation of chronic p38 mitogen-activated protein kinase (MAPK) and a lower level of the polycomb protein Bmi-1. However, the mechanism underlying Bmi-1 down-regulation and its relevance to defective proliferation in *Atm^-/-^* NSCs remained unclear. Here, we show that over-expression of Bmi-1 increases self-renewal and proliferation of *Atm^-/-^* NSCs to normal, indicating that defective proliferation in *Atm^-/-^* NSCs is a consequence of down-regulation of Bmi-1. We also demonstrate that epidermal growth factor (EGF)-induced Akt phosphorylation renders Bmi-1 resistant to the proteasomal degradation, leading to its stabilization and accumulation in the nucleus. However, inhibition of the Akt-dependent Bmi-1 stabilizing process by p38 MAPK signaling reduces the levels of Bmi-1. Treatment of the *Atm^-/-^* NSCs with a specific p38 MAPK inhibitor SB203580 extended Bmi-1 posttranscriptional turnover and H2A ubiquitination in *Atm^-/-^* NSCs. Our observations demonstrate the molecular basis underlying the impairment of self-renewal and proliferation in *Atm^-/-^* NSCs through the p38 MAPK-Akt-Bmi-1-p21 signaling pathway.

## Introduction

ATM (A-T mutated) kinase plays a critical role in the regulation of cell cycling, DNA repair, and cellular redox status. ATM expression is abundant in neural stem cells (NSCs), but is gradually reduced as the cell differentiates [1], suggesting that ATM may play an essential role in NSC survival and function. In normal brain tissue, the number of NSCs is the result of a tightly controlled balance between self-renewal, proliferation, differentiation and death. This means that proper control of these events is critical for maintaining a normal numbers of neurons, astrocytes, and oligodendrocytes in the brain [2]. The hypothesis that NSCs are functionally defective in the absence of ATM has not been conclusively determined. Abnormal neuronal and astrocytic development was reported in ATM knockout mice [1], [3]–[5], which could be the result of abnormal differentiation of NSCs. We previously reported that ATM is required to maintain normal self-renewal and proliferation of NSCs, due to its role in controlling the redox status. Loss of ATM renders NSCs defective in proliferation through oxidative stress-dependent p38 MAPK (hereafter called p38) signaling [6]. However, despite the mounting interest, the mechanisms regulating NSC survival and proliferation in response to oxidative stress remain elusive in this mouse model.

In the present study we found that Bmi-1 is highly downregulated in Atm^-/-^ NSCs, or when normal NSCs are treated with H_2_O_2_. Bmi-1 functions as a component of the polycomb repressive protein complex [7]–[8]. It interacts with RING1B, another component of polycomb repressive protein complex 1, causing H2A ubiquitination and modification of chromatin structure leading to epigenetically suppress gene expression [9]. In NSCs, particularly those derived from the SVZ and hippocampus, Bmi-1 is necessary for normal NSC self-renewal and survival, because it silences genes that encode the cell cycle inhibitors p16, p19, and p21 [8], [10]–[11]. Accordingly, shRNA knockdown of *bmi*-*1* results in up-regulation of p21, which in turn causes suppression of NSC self-renewal and proliferation [11]. Interestingly, Bmi-1 can also separately regulate mitochondrial function [12] and redox homeostasis by reducing the intracellular levels of ROS [13]. Thus, down-regulation of Bmi-1 in cells results in oxidative stress, just as ATM deficiency does. Furthermore, Bmi-1 deficient mice resemble *Atm^-/-^* mice, as they exhibit oxidative stress and a progressive postnatal depletion of NSCs that leads to neurological abnormalities and ataxia [11]–[12]. Therefore, further investigation into the mechanisms underlying Bmi-1 down-regulation in *Atm^-/-^* NSCs is necessary to better understand the role of ATM in regulating self-renewal and proliferation of NSCs.

Until now, the identity of upstream effectors that control the level or the function of Bmi-1 remains unclear. Here we show that Bmi-1 may be a substrate of Akt and up-regulation of Akt signaling coincides with up-regulation of Bmi-1 phosphorylation and that phosphorylated Bmi-1 is more stable. In contrast, activated oxidative stress-dependent p38 signaling causes Bmi-1 to degrade, and lose its chromatin modifying ability. Furthermore, we also show that posttranscriptional regulation of Bmi-1 via proteasomal degradation causes defective proliferation in *Atm^-/-^* NSCs as a result of p21 up-regulation. Collectively, these observations strongly support the notion that down-regulation of Bmi-1 is associated with oxidative stress-dependent pathways in *Atm^-/-^* NSCs with p38 activation, Akt deactivation, and p21 up-regulation. Our findings have identified these players as potential targets for A-T treatment.

## Results

### Bmi-1 is posttranscriptionally downregulated in *Atm^-/-^* NSCs

**Figure 1:**
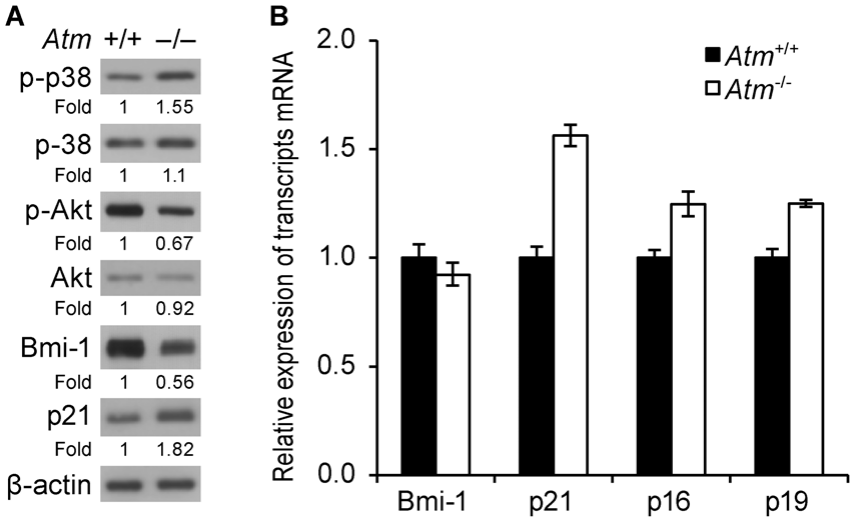
Bmi-1 is posttranscriptionally downregulated in *Atm^-/-^* NSCs. (A) Protein samples (30 µg) from *Atm* ^+/+^ and *Atm^-/-^* NSCs were analyzed by Western blotting for phospho-p38, p38, phospho-Akt, Akt, Bmi-1, and p21 expression. (B) Total RNA was purified from *Atm* ^+/+^ and *Atm^-/-^* NSCs and subjected to quantitative RT-PCR analysis for *bmi-1*, *p21*, *p16*, and *p19* expression. *Gapdh* was used as an internal control.

We previously reported that *Atm^-/-^* NSCs display highly decreased self-renewal and proliferation, which is correlated with increased levels of CDK inhibitors [6]. The polycomb protein Bmi-1 promotes NSC self-renewal and proliferation through the repression of CDK inhibitor genes [8], [10]–[11], therefore we investigated Bmi-1 levels in *Atm^-/-^* NSCs. *Atm^-/-^* NSCs show decreased levels of Bmi-1 and Akt activation, which are essential for NSC proliferation. These results are accompanied by elevated levels of activated-p38 and p21 (Figure 1A). To investigate whether the loss of ATM affected *bmi-1* expression in NSCs, quantitative RT-PCR analysis was performed to measure mRNA levels. No significant decrease of *bmi-1* mRNA were seen in *Atm^-/-^* NSCs (Figure 1B). We also examined the mRNA levels of the CDK inhibitors *p21*, *p16*, and *p19*, which are regulated by Bmi-1, and observed elevated levels of these transcripts in *Atm^-/-^* NSCs. This observation is consistent with studies by others that *p21*, *p16*, and *p19* expression levels are increased in *bmi-1^-/-^* NSCs [8], [10] or in *bmi-1*-knockdown NSCs [11]. The magnitude of increase in *p21* mRNA was higher than the increase seen in the mRNA levels of *p16* and *p19* (Figure 1B). Using fluorescence microscopy, we confirmed that levels of activated p38 and p21 are up-regulated, whereas levels of activated Akt and Bmi-1 are downregulated in *Atm^-/-^* NSCs (Figure S1). These results indicate that the loss of ATM causes a down-regulation in Bmi-1 protein levels in NSCs posttranscriptionally.

### Down-regulation of Bmi-1 causes defective proliferation in *Atm^-/-^* NSCs

*Atm^-/-^* NSCs exhibit significantly reduced proliferation and Bmi-1 is downregulated in these cells.

**Figure 2:**
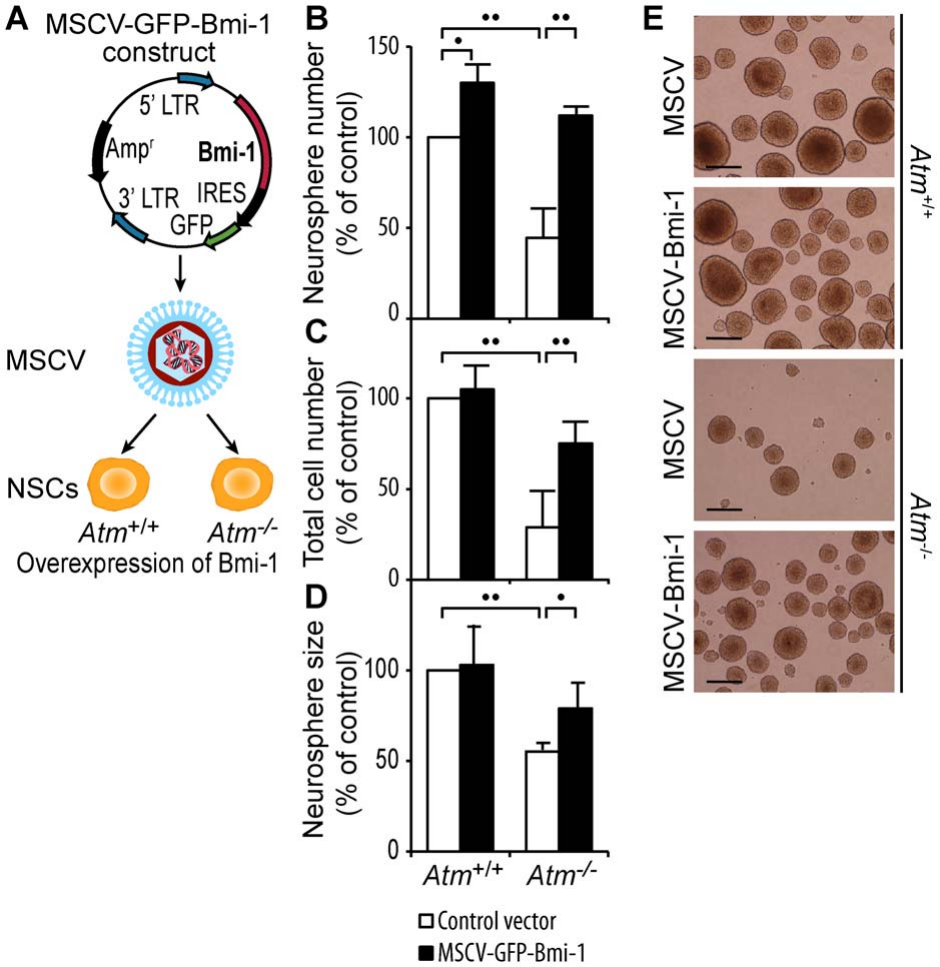
Down-regulation of Bmi-1 causes defective proliferation in *Atm^-/-^* NSCs. (A) Generation of Bmi-1 over-expressing NSCs by infecting *Atm* ^+/+^ or *Atm^-/-^* NSCs with MSCV-GFP-bmi-1 bearing retrovirus. (B) Neurosphere number generated by control and Bmi-1 over-expressing *Atm* ^+/+^ and *Atm^-/-^* NSCs. (C) Total cell number per well generated by control and Bmi-1 over-expressing *Atm* ^+/+^ and *Atm^-/-^* NSCs. (D) Neurosphere size generated by control and Bmi-1 over-expressing *Atm* ^+/+^ and *Atm^-/-^* NSCs. (B-D) The mean±S.E. of three independent experiments is shown. ^•^ *p*<0.05; ^••^ *p*<0.01, when GFP-Bmi-1 expressing *Atm* ^+/+^ NSCs were compared with GFP expressing *Atm* ^+/+^ NSCs, when GFP-Bmi-1 expressing *Atm^-/-^* NSCs were compared with GFP expressing *Atm^-/-^* NSCs, or when GFP expressing *Atm^-/-^* NSCs were compared with GFP expressing *Atm* ^+/+^ NSCs. (E) Phase-contrast photomicrographs of *Atm* ^+/+^ and *Atm^-/-^* neurospheres, either expressing MSCV-GFP or MSCV-GFP-Bmi-1. Scale bars  = 50 µm.

Therefore, we decided to investigate whether down-regulation of Bmi-1 contributes to defective proliferation of *Atm^-/-^* NSCs. To address this question, we generated Bmi-1 over-expressing *Atm* ^+/+^ and *Atm^-/-^* NSCs by infecting cells with a retrovirus bearing Bmi-1. A retrovirus bearing Bmi-1 was made using a mouse stem cell virus (MSCV) vector, which contains an internal ribosome entry site (IRES) followed by GFP (Figure 2A) [14].

To compare the proliferative capacity of control *vs*. Bmi-1 over-expressing NSCs, the number of new neurospheres, total number of cells per well, and the size of neurospheres generated 2 days after subculture were determined. The number of newly generated neurosphere is indicative of NSC self-renewing activity, total cell number in a well represents mitotic division of daughter cells in neurosphere, and the size of newly generated neurosphere represents proliferation within each neurosphere. As expected and confirming the observation from our previous study [6], *Atm^-/-^* NSCs formed significantly lower neurosphere numbers, total cell numbers per well, and smaller size of neurospheres compared to *Atm* ^+/+^ NSCs. However, Bmi-1 over-expression by infecting *Atm^-/-^* NSCs with a retrovirus bearing Bmi-1 greatly improved newly formed neurosphere numbers (Figure 2B), total cell numbers per well (Figure 2C), and neurosphere size (Figure 2D). Neurosphere number of *Atm^-/-^* NSCs over-expressing Bmi-1 was comparable to untreated *Atm* ^+/+^ NSCs, while neurosphere size and total cell number were partially recovered. We also observed that Bmi-1 over-expression increased proliferation in *Atm* ^+/+^ NSCs by increase in neurosphere numbers.

In figure 2E, photomicrographs of both *Atm* ^+/+^ and *Atm^-/-^* neurospheres expressing either MSCV-GFP or MSCV-GFP-Bmi-1 are shown. The restorative action of Bmi-1 over-expression was evident in floating neurospheres, as shown by increased neurosphere number and size in *Atm^-/-^* NSCs. Collectively, these results demonstrate that NSCs require Bmi-1 for survival and normal proliferation, and that down-regulation of Bmi-1 substantially contributes to the defective proliferation of *Atm^-/-^* NSCs.

### Bmi-1 undergoes proteasome-mediated degradation, which is accelerated during oxidative stress

**Figure 3:**
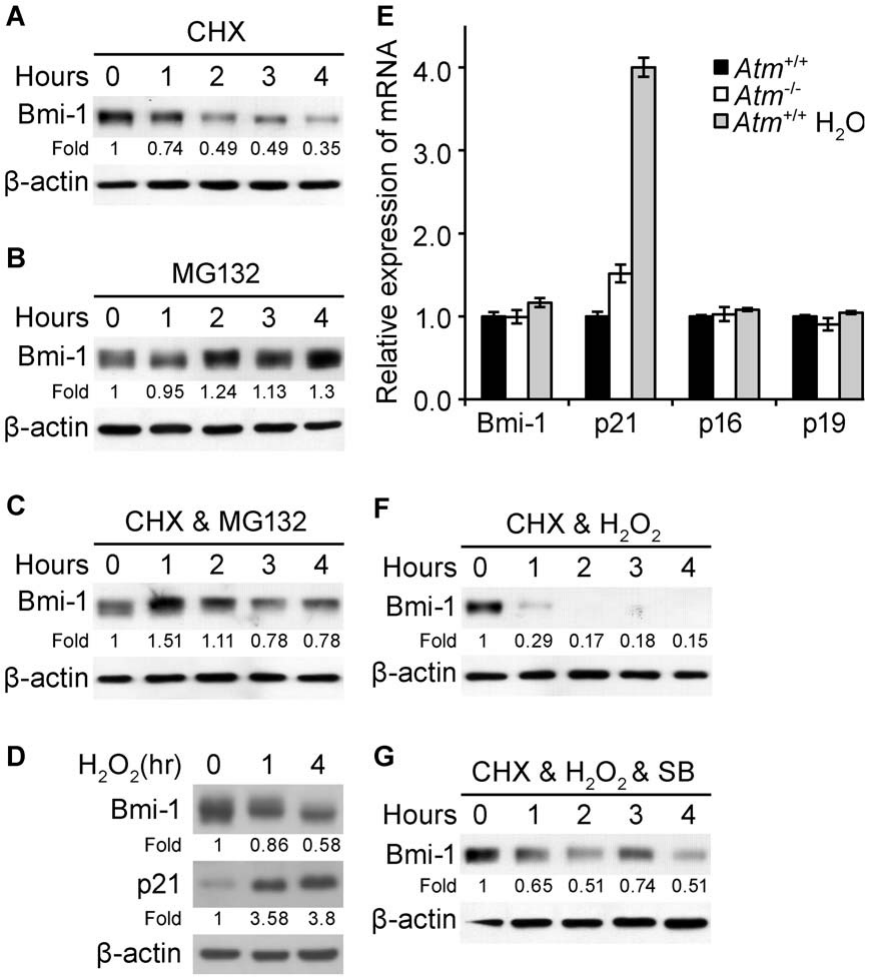
Bmi-1 undergoes proteasome-mediated degradation, which is accelerated during oxidative stress. (A) *Atm* ^+/+^ NSCs were treated for the times indicated with cycloheximide (50 µg/mL) and Bmi-1 levels were determined by Western blotting. Probing with an antibody to β-actin was used as a loading control. The values represent the Bmi-1 signal intensity compared to the 0 h time point. (B) *Atm* ^+/+^ NSCs were treated for the times indicated with MG132 (10 nM) and Bmi-1 levels were determined by Western blotting. Probing with an antibody to β-actin was used as a loading control. The values represent the Bmi-1 signal intensity compared to the 0 h time point. (C) *Atm* ^+/+^ NSCs were co-treated for the times indicated with cycloheximide (50 µg/mL) and MG132 (10 nM), and Bmi-1 levels were determined by Western blotting. Probing with an antibody to β-actin was used as a loading control. The values represent the Bmi-1 signal intensity compared to the 0 h time point. (D) NSCs from *Atm* ^+/+^ mice were treated for the times indicated with 100 µM H_2_O_2_ with the addition of MG132 (10 nM), and Bmi-1 and p21 levels were determined by Western blotting. (E) NSCs from *Atm* ^+/+^ or *Atm^-/-^* mice were treated for 7 hours with 100 µM H_2_O_2_ and total RNA was purified and analyzed by quantitative RT-PCR analysis for *bmi-1, p21, p16, and p19* expression. Probing for *gapdh* was used as an internal control. (F) NSCs from *Atm* ^+/+^ mice were treated for the times indicated with cycloheximide (50 µg/mL) and 100 µM H_2_O_2_, and Bmi-1 levels were determined by Western blotting. Probing with an antibody to β-actin was used as a loading control. The values represent the Bmi-1 signal intensity compared to the 0 h time point. (G) NSCs from *Atm* ^+/+^ mice were pretreated with 10 µM SB203580 for 2 hours before treated for the times indicated with cycloheximide (50 µg/mL), and Bmi-1 levels were determined by Western blotting. Probing with an antibody to β-actin was used as a loading control. The values represent the Bmi-1 signal intensity compared to the 0 h time point.

Other researchers demonstrated that Bmi-1 expression is modulated through transcriptional and posttranscriptional regulation in hematopoietic stem cells [15]. Here, we observed no distinct differences in *bmi-1* mRNA levels between *Atm* ^+/+^ and *Atm* ^-/-^ NSCs, although decreased level of Bmi-1 protein was noted in *Atm* ^-/-^ NSCs (Figure 1). These results indicate that Bmi-1 may be downregulated via a posttranscriptional mechanism in *Atm^-/-^* NSCs. To determine if the level of Bmi-1 is regulated via posttranscriptional proteasome-mediated degradation in NSCs, we examined Bmi-1 stability in *Atm* ^+/+^ NSCs treated with the protein synthesis inhibitor cycloheximide. Cycloheximide-treated cells showed a rapid decline in Bmi-1 levels with half-life about 2 hours (Figure 3A). To investigate whether proteasome inhibition affects the levels of endogenous Bmi-1, NSCs were treated with proteasome inhibitor MG132. Treatment of cells with MG-132 significantly increased Bmi-1 levels compared to those in untreated cells (Figure 3B). In addition, co-treatment with MG-132 and cycloheximide slowed the degradation of Bmi-1, compared to single treatment with cycloheximide (Figure 3C). These results indicate that Bmi-1 turn over can be antagonized by proteasome inhibition.

We have previously demonstrated that excess ROS elicited by H_2_O_2_ treatment decreased NSC proliferation through activation of p38 signaling, similar to that in *Atm* ^-/-^ NSCs [6]. Therefore, to ascertain whether oxidative stress could also affect Bmi-1 degradation, we treated *Atm* ^+/+^ NSCs with H_2_O_2_ and analyzed Bmi-1 and p21 levels. Western blot analysis revealed that Bmi-1 appears as a triplet of approximately 40 to 44-kDa. Currently, no specific antibody is available to detect the phosphorylated form of Bmi-1, thus phosphorylation is determined by mobility shift. After 1 hour of treatment with H_2_O_2_, the unphosphorylated Bmi-1 band was significantly reduced (faster migration on gel), resulting in an increase in the phosphorylated bands (slower migration on gel), compared to those of untreated samples. After 4 hours of incubation with H_2_O_2_, we observed a dramatic reduction in the level of phosphorylated Bmi-1 (decreased by 90% compared to the untreated sample), as well as a significant reduction in the level of Bmi-1 (Figure 3D). In addition, H_2_O_2_ caused a significant increase in p21 protein levels in NSCs. Interestingly, H_2_O_2_ selectively caused a significant elevation in *p21* expression, whereas expression of *bm*i-1, *p16*, and *p19* were not altered in H_2_O_2_-treated NSCs (Figure 3 E). The reason of the selectivity of *p21* expression by oxidative stress is unclear. However, it can be explained by the previous study demonstrating that p21 protects cells from oxidative damage through activation of Nrf2 as an antioxidant response [16], and it was also shown that Bmi-1 is important for NSC proliferation and survival at earlier embryonic stages, via suppression of cell cycle inhibitor p21, potentially with stage-related differences [11].

The result as shown above that Bmi-1 is posttranscriptionally downregulated in response to H_2_O_2_ (Figure 3D and E) prompted us to examine whether oxidative stress causes a change in Bmi-1 turnover. Wild-type NSCs were co-treated with H_2_O_2_ and cycloheximide. We observed that H_2_O_2_ dramatically shortens Bmi-1 half-life (Figure 3F), whereas the p38 inhibitor SB203580 slowed this rapid degradation of Bmi-1 (Figure 3G), suggesting that the H_2_O_2_-induced rapid degradation of Bmi-1 is a p38-dependent pathway. This finding is consistent with our previous observation that oxidative stress reduces proliferation of NSCs through p38 signaling [6]. Collectively, these results demonstrate that Bmi-1 undergoes proteasome-mediated degradation, and that oxidative stress elicited by H_2_O_2_ accelerates Bmi-1 degradation through p38 signaling in NSCs.

### EGF-mediated Akt signaling causes Bmi-1 phosphorylation and up-regulation in NSCs

**Figure 4:**
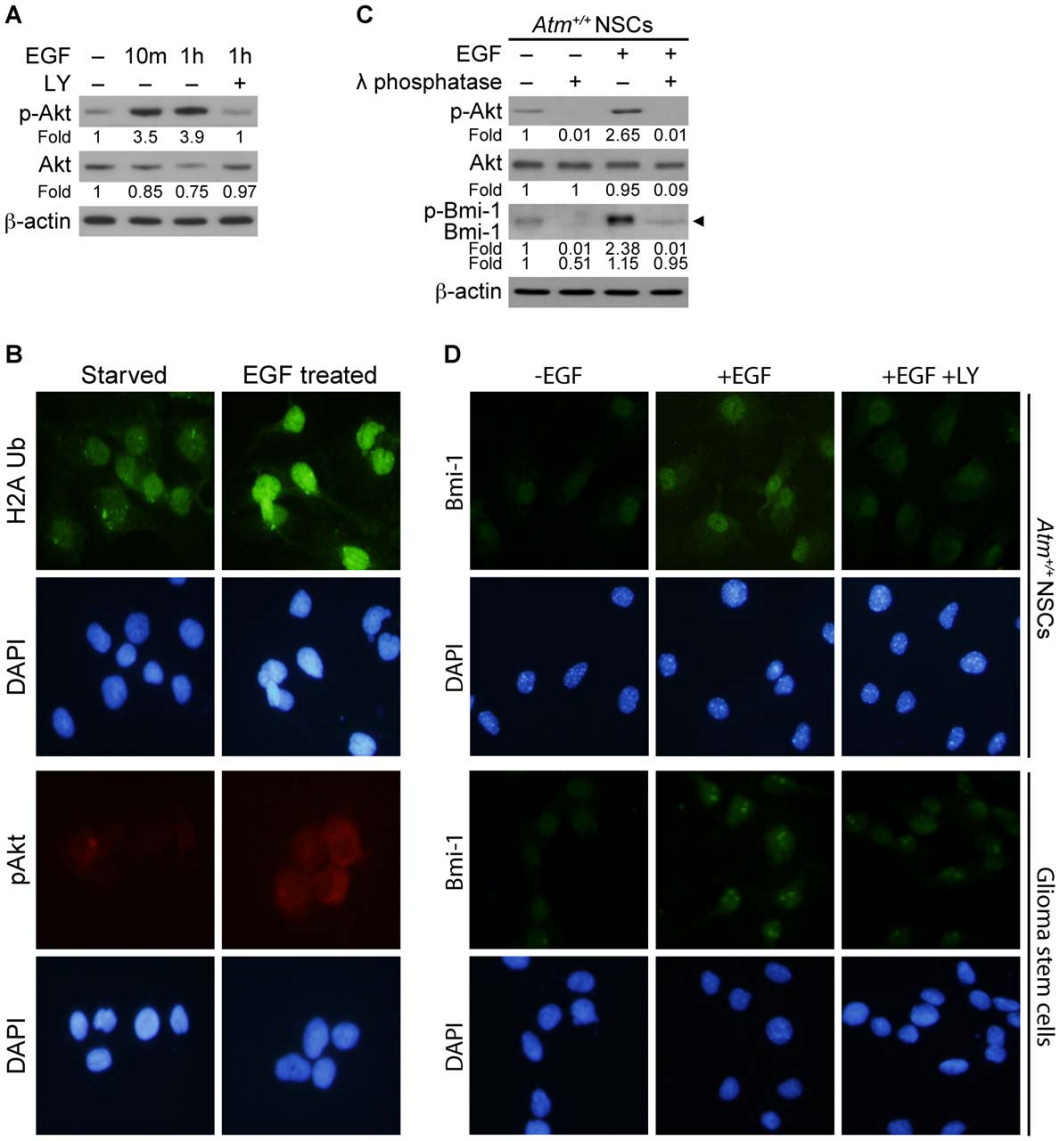
EGF-mediated Akt signaling causes Bmi-1 phosphorylation and up-regulation in NSCs. (A) *Atm* ^+/+^ NSCs were deprived of epidermal growth factor (EGF, 20 ng/ml) for 4 hours, at which time EGF was added back to the cultures for indicated times, with or without addition of LY2949002 (LY, 10 µM), and analyzed by Western blotting for phospho-Akt and Akt levels. (B) After EGF starvation of *Atm* ^+/+^ NSCs for 4 hours, EGF was added back to the cultures for 3 hours. Neurospheres were analyzed by Immunocytochemistry for the levels of ubiqutinated-histone H2A (H2A-Ub) and phospho-Akt. Cells were counterstained by DAPI (4′-6-Diamidino-2-phenylindole), which identifies the nuclei of the NSCs. (C) After EGF starvation of *Atm* ^+/+^ NSCs for 4 hours, EGF was added back to the cultures for 3 hours and analyzed for levels of phospho-Akt, Akt, and Bmi-1 by Western blotting. Protein samples (30 µg) were then treated in vitro with λ phosphatase (Ser/Thr and Tyr phosphatase) for 1.5 hours and analyzed for changes in migration of Bmi-1 in SDS-PAGE. Arrowhead indicates totally dephosphorylated forms of Bmi-1 that can be used as the reference band to visualize the mobility shift of Bmi-1 (faster migration on gel). (D) *Atm* ^+/+^ NSCs and GSCs were deprived of EGF for 4 hours. EGF was added back to the cultures for 3 hours, with or without LY2949002. Bm-1 levels and localization were analyzed by Immunofluorescence (FITC-green). Cells were counterstained by DAPI (4′-6-Diamidino-2-phenylindole), which identifies the nuclei of the NSCs.

Neurosphere formation and NSC proliferation are dependent on EGF (Epidermal growth factor) through signaling by the EGFR (Epidermal Growth Factor Receptor) tyrosine kinase [17]. Therefore, we investigated if signaling by EGF had functional consequences on the proteasomal degradation of Bmi-1. First, the phosphorylation state of Akt was analyzed to evaluate the activity of the phosphoinositide-3-kinase (PI3-K) signaling pathway after EGF treatment. We observed that Akt is activated shortly after EGF treatment in EGF-starved *Atm* ^+/+^ NSCs and activation is inhibited by PI3-K inhibitor, LY2949002 (Figure 4A). Because Bmi-1 represses gene expression of CDK inhibitors through H2A ubiquitination and promotes NSC proliferation [18]–[19], we examined the effects of EGF treatment on H2A ubiquitination. H2A ubiquitination was reduced during EGF-starvation, but was restored when EGF was added back to the culture (Figure 4B) coincided with Akt activation, indicating that H2A ubiquitination by Bmi-1 requires EGF-Akt signaling. Next, we examined whether EGF-Akt signaling affects Bmi-1 levels and found that Akt activation by EGF treatment coincided with Bmi-1 up-regulation. We also investigated if Bmi-1 up-regulation by EGF correlates with phosphorylation by Akt. To verify the phosphorylation status of Bmi-1, we treated the protein extracts with λ phosphatase, a Ser/Thr and Tyr phosphatase. We found that phosphorylation of Bmi-1 (slower migration on the gel) was increased by EGF treatment and was eliminated by λ phosphatase as evidenced by faster migration on the gel (Figure 4C), indicating that Bmi-1 is a phosphoprotein. Collectively, these results imply that Bmi-1 up-regulation and its function in histone H2A ubiquitination is dependent on Akt signaling in NSCs. We next questioned whether Akt is involved in Bmi-1 phosphorylation, thus we performed an amino acid sequence analysis to identify Bmi-1 phosphorylation sites (http://www.scansite.edu). Since Bmi-1 contains a predicted Akt substrate motif, we decided to determine if Bmi-1 is a substrate for Akt. Bmi-1 purified by immunoprecipitation from NSCs was detected by anti-phospho (Ser/Thr) Akt substrate antibody. However, it was less detectable by anti-phospho (Ser/Thr) Akt substrate antibody in NSCs treated with LY294002 (data not shown), suggesting that Bmi-1 may be a substrate of Akt.

In *Atm* ^+/+^ NSCs, Bmi-1 is highly expressed and localizes in the nucleus. Bmi-1 levels were reduced under EGF-starvation conditions or after treatment with LY2949002 (Figure 4D, upper panels), further suggesting Akt-dependent Bmi-1 up-regulation. In the presence of EGF stimulus, Bmi-1 localizes in chromatin of glioma stem cells (GSCs), evidenced by the fact that Bmi-1 in GSCs co-localizes with histone H3 in the immunocytochemistry image (data not shown). However, EGF-starvation significantly abolishes Bmi-1 nuclear accumulation and the LY2949002 partially blocks Bmi-1 nuclear accumulation (Figure 4D, lower panels). These results suggest that Bmi-1 nuclear localization is also an Akt-dependent process. Our results demonstrate that Akt signaling causes Bmi-1 phosphorylation in NSCs resulting in up-regulation of Bmi-1.

### Bmi-1 is downregulated by p38 signaling during oxidative stress through Akt inhibition

**Figure 5:**
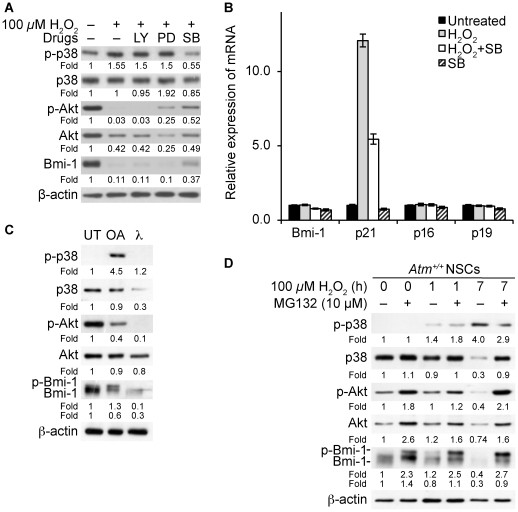
Bmi-1 is downregulated by p38 signaling during oxidative stress through inhibiting Akt. (A) *Atm* ^+/+^ NSCs were pretreated for 2 hours with either LY2949002 (LY, 10 µM), PD98059 (PD, 10 µM), or SB203580 (SB, 10 µM), and then treated for 4 hours with 100 µM H_2_O_2_. Protein samples were analyzed by Western blotting fo levels of phospho-p38, p38, phospho-Akt, Akt, and Bmi-1. (B) *Atm* ^+/+^ NSCs were pretreated for 2 hours with SB203580 (SB, 10 µM) and then treated for 4 hours with 100 µM H_2_O_2_. Total RNA were purified and analyzed by quantitative RT-PCR analysis for *bmi-1, p21, p16, and p19* expression. Probing for *gapdh* was used as an internal control. (C) *Atm* ^+/+^ NSCs were treated for 4 hours with okadaic acid (OA, PP2A inhibitor) or 100 µM H_2_O_2_. Protein samples (30 µg) were treated in vitro with λ phosphatase for 1.5 hours and analyzed by Western blotting for levels of phospho-p38 and p38, phospho-Akt, Akt, and Bmi-1. (D) *Atm* ^+/+^ NSCs were treated with 100 µM H_2_O_2_ for times indicated in the presence or absence of 10 µM MG132, and analyzed by Western blotting for levels of phospho-p38 and p38, phospho-Akt, Akt, and Bmi-1.

We have previously reported that H_2_O_2_ highly decreases NSC proliferation, and that the p38 inhibitor, SB203580, restores normal proliferation [6]. In the present study we showed that H_2_O_2_ decreased Bmi-1 levels in NSCs (Figure 3D), therefore we asked whether the inhibition of p38 signaling by SB203580 rescues Bmi-1 levels even under oxidative stress conditions. We observed that H_2_O_2_ down-regulates Bmi-1 in *Atm* ^+/+^ NSCs, but SB203580 to some extent restores Bmi-1 levels in H_2_O_2_-treated NSCs. Restorative effects were not observed after treatment with the PI3-K inhibitor LY2949002, nor ERK1/2 inhibitor PD98059 (Figure 5A). Since *p21* expression was selectively increased by H_2_O_2_-treatment in *Atm* ^+/+^ NSCs (Figure 3E), we also questioned whether p38 inhibition by SB203580 has an effect on *p21* expression in H_2_O_2_-treated NSCs. As expected, no distinct differences in *bmi-1*, *p16*, and *p19* expression were observed in H_2_O_2_-treated NSCs. *p21* expression significantly increased in response to H_2_O_2_ treatment, but the p38 inhibitor, SB203580, highly reduced *p21* expression (Figure 5B), further indicating that during oxidative stress Bmi-1 down-regulation and its functional consequence are a p38 dependent process.

The existence of cross talk between p38 MAPKs and the PI3K/Akt and/or ERK survival pathways has also been described in other cellular systems. For example, activation of p38 MAPKs inhibits the PI3K/Akt and/or ERK pathways through activation of PP2A phosphatases [20]–[22]. In the present study, we showed Akt pathway stabilizes Bmi-1 (Figure 4). However, negative regulation of Akt by p38 in NSCs and the relevance of this cross talk in the Bmi-1 regulation are unclear. Therefore, we decided to investigate whether negative regulation of Bmi-1 by p38 could be linked to Akt and/or PP2A regulation. We treated *Atm* ^+/+^ NSCs with okadaic acid, a PP2A inhibitor or with λ phosphatase, a Ser/Thr and Tyr phosphatase. We expected that okadaic acid would increase Akt phosphorylation because PP2A is known to dephosphorylate Akt. However we observed that okadaic acid decreased Akt phosphorylation levels. In contrast, okadaic acid triggered phosphorylation of both p38 and Bmi-1. This result indicates that p38 negatively regulates Akt via PP2A in NSCs, and that Bmi-1 is phosphorylated by an Akt-independent mechanism. On the other hand, treatment of protein extract with λ phosphatase dephosphorylated p38 and Akt, as well as Bmi-1 (Figure 5C). These results suggest that there might be an inhibitory cross talk between p38 and Akt as they show opposite activation events by okadaic acid and implies the relevance of Akt inhibition by PP2A in the down-regulation of Bmi-1 induced by p38 activation.

Interestingly, okadaic acid treatment has similar effects on p38, Akt, and Bmi-1 with H_2_O_2_ treatment, eliciting p38 and Bmi-1 phosphorylation and Akt dephosphorylation (compare with Figure 5D, at 1 hour). These results suggest that although Bmi-1 contains no known p38 phosphorylation motif (http://www.scansite.edu), p38 may also phosphorylate Bmi-1 when Akt is unable to phosphorylate Bmi-1 under oxidative stress conditions. To further link p38/Akt to Bmi-1 stability, we asked whether the proteasome inhibitor MG132 prevents H_2_O_2_-induced p38 phosphorylation and restores levels of Akt phosphorylation and Bmi-1 expression. We treated *Atm* ^+/+^ NSCs with H_2_O_2_ for 1 or 7 hours. Bmi-1 levels were decreased at 1 hour and barely detected at 7 hours. In contrast, MG132 stabilized Akt phosphorylation, coinciding with Bmi-1 up-regulation and phosphorylation (Figure 5D). These observations indicate that the Akt-dependent phosphorylation of Bmi-1 results in its up-regulation by inhibiting the proteasome-dependent degradation process. Interestingly, MG132 downregulates p38 activation levels (Figure 5D) and SB203580 restored Bmi-1 levels in H_2_O_2_-treated *Atm* ^+/+^ NSCs (Figure 5A). Collectively, these results demonstrate that during oxidative stress, Bmi-1 is downregulated by p38-mediated proteasomal degradation through Akt inhibition.

### p38 signaling leads to proteasomal degradation of Bmi-1 in *Atm^-/-^* NSCs

**Figure 6:**
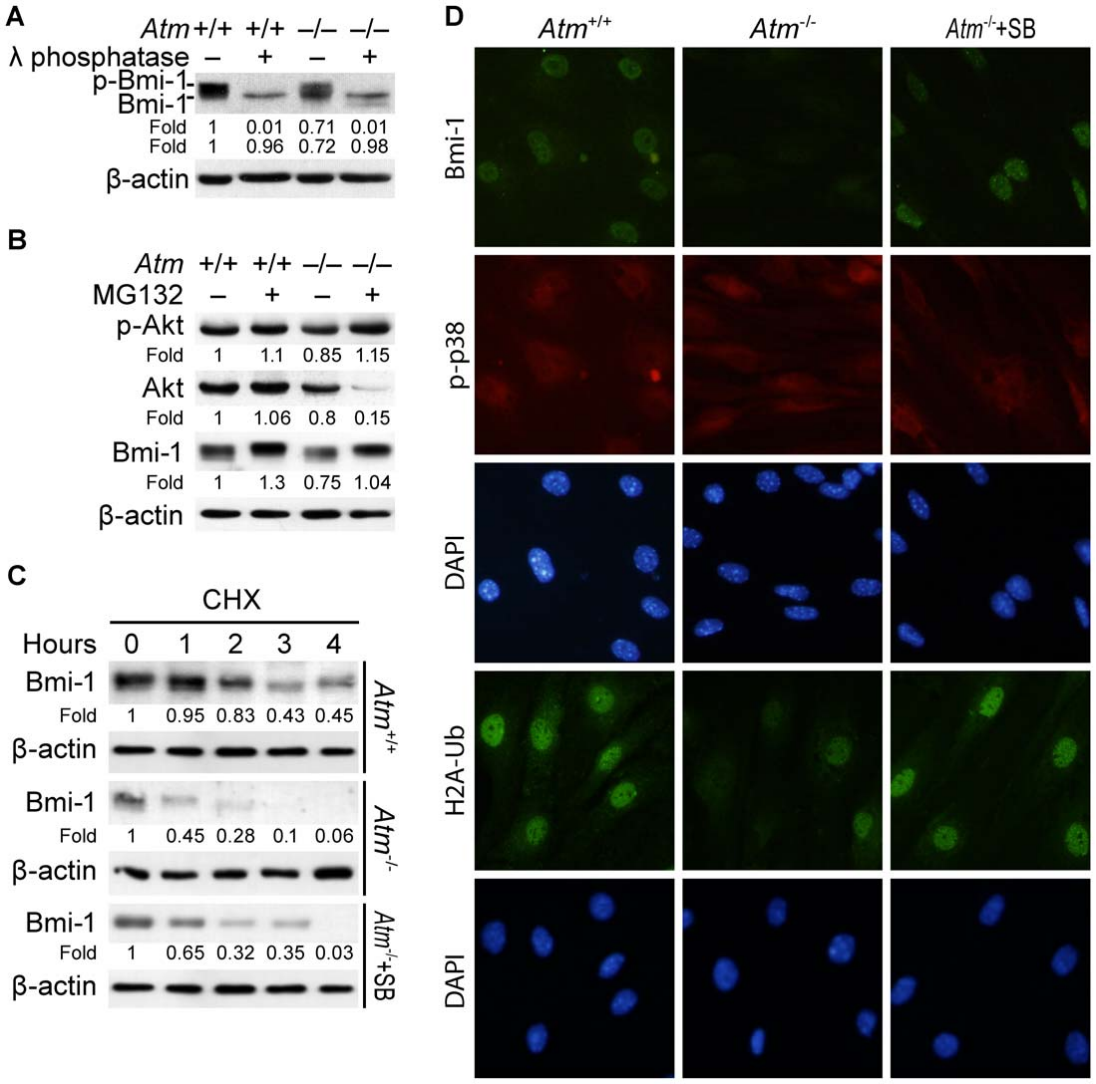
p38 signaling leads to proteasomal degradation of Bmi-1 in *Atm^-/-^* NSCs. (A) Protein samples (30 µg) from *Atm* ^+/+^ or *Atm^-/-^* NSCs were treated in vitro with λ phosphatase for 1.5 hours and analyzed by Western blotting for changes in migration of Bmi-1 in SDS-PAGE. (B) *Atm* ^+/+^ or *Atm^-/-^* NSCs were treated for 4 hours with 10 µM MG132 and phospho-Akt, Akt, and Bmi-1 levels were determined by Western blotting. (C) *Atm^-/-^* NSCs were pretreated with 10 µM SB203580. *Atm* ^+/+^ and *Atm^−/−^* NSCs were then treated for times indicated with cycloheximide (50 µg/mL) and Bmi-1 levels were determined by Western blotting. Probing with an antibody to β-actin was used as a loading control. The values represent the Bmi-1 signal intensity compared to the 0 h time point. (D) *Atm* ^+/+^ or *Atm^-/-^* NSCs treated for 24 hours with either cultured in simple medium (middle panel) or 10 µM SB203580 (right panel) were analyzed by Immunofluorescence for Bmi-1, phospho-p38, and ubiqutinated-histone H2A (H2A-Ub). Cells were counterstained by DAPI (4′-6-Diamidino-2-phenylindole), which identifies the nuclei of the NSCs.

We compared the phosphorylation status of Bmi-1 in protein extracts from *Atm* ^+/+^ and *Atm^-/-^* NSCs after treatment with λ phosphatase. Bmi-1 phosphorylation was unimpaired regardless of the status of ATM, although total levels of Bmi-1 decreased in *Atm^-/-^* NSCs (Figure 6A). This phenomenon might be explained by the fact that under normal conditions Akt is primarily responsible for phosphorylating Bmi-1, but in *Atm^-/-^* NSCs, p38 is still capable of phosphorylating Bmi-1 in an ATM-independent manner. Next, we sought to determine whether up-regulation of Bmi-1 and Akt signaling correlated with inhibition of Bmi-1 proteasomal degradation. We treated *Atm* ^+/+^ and *Atm^-/-^* NSCs with MG132 for 4 hours to monitor Akt activation and Bmi-1 levels. We found that MG132 treatment increased Akt activation and Bmi-1 levels in both cells, and that Akt activation by MG132 treatment was accompanied by increased Bmi-1 levels. In addition, the levels of Akt activation and Bmi-1 expression in *Atm^-/-^* NSCs treated with MG132 were comparable to levels in MG132-treated *Atm* ^+/+^ NSCs (Figure 6B). These results suggest that Bmi-1 down-regulation in NSCs occurs by proteasomal degradation and Akt signaling protects Bmi-1 from proteasomal degradation.

We analyzed an Akt activation profile in *Atm* ^+/+^ and *Atm^-/-^* NSCs after exposure to H_2_O_2_. We found that *Atm^-/-^* NSCs, but not *Atm* ^+/+^ NSCs, failed to activate Akt after exposure to H_2_O_2_ (data not shown), demonstrating that ATM is required for the activation of Akt. In the absence of ATM, Bmi-1 phosphorylation by Akt is reduced, which might accelerate the p38-dependent degradation of Bmi-1. Notably, treatment of *Atm^-/-^* NSCs with SB203580 restored Akt activation and normal proliferation [6], which implies that in *Atm^-/-^* NSCs, Bmi-1 degradation may be accelerated by chronic p38 activation. To address this hypothesis we treated *Atm* ^+/+^ and *Atm^-/-^* NSCs with cycloheximide, to block de novo synthesis of Bmi-1 and the half-life of Bmi-1 was compared by Western blotting analysis. Next, to confirm that Bmi-1 degradation is linked to p38 signaling in *Atm* ^-/-^ NSCs, we treated *Atm* ^-/-^ NSCs with SB203580 and calculated the half-life of Bmi-1 as compared to untreated NSCs. We observed that the half-life of Bmi-1 in *Atm^-/-^* NSCs was shortened compared to *Atm* ^+/+^ NSCs, but SB203580 extended the turnover of Bmi-1 in *Atm^-/-^* NSCs (Figure 6C). We also found that Bmi-1 levels and H2A ubiquitination are reduced in *Atm^-/-^* NSCs compared to *Atm* ^+/+^ NSCs. Inhibition of p38 signaling by SB203580 significantly restored these levels in *Atm^-/-^* NSCs (Figure 6D). Taken together with the up-regulation of p38 signaling and the down-regulation of Akt signaling in *Atm^-/-^* NSCs (Figure 1A), these results demonstrate that in *Atm^-/-^* NSCs p38-mediated proteasomal degradation of Bmi-1 is accelerated compared to normal NSCs.

## Discussion

The majority of previous investigations examining A-T associated neurodegeneration have focused on neurons and astrocytes. In the present study we focused on NSCs. NSC-specific targeting holds the prospect to provide a cure for many neurodegenerative diseases, as NSCs possess the potential to differentiate into neurons, oligodendrocytes, and astrocytes. Because of their pluripotency, impairment of NSCs likely results in neuronal cell loss in *Atm^-/-^* mice. We previously demonstrated that NSCs from SVZ of *Atm^-/-^* mouse brain are abnormal, in terms of decreased proliferative capacity and lower Bmi-1 expression levels compared to *Atm* ^+/+^ NSCs [6].

Here we show that down-regulation of Bmi-1 contributes to defective self-renewal and proliferation in *Atm^-/-^* NSCs because over-expression of Bmi-1 restores *Atm^-/-^* NSC proliferation to normal (Figure 2). Additionally, we identified upstream effectors that control Bmi-1 levels and function in chromatin modification. Our data suggests that p38 is a central player in the defective proliferation of *Atm^-/-^* NSCs induced by oxidative stress. Previous report demonstrated that p38 participates in p21 up-regulation, subsequently resulting in cell growth inhibition [23]. Here, we show that Bmi-1 levels are regulated post-transcriptionally by proteasome-mediated degradation (Figure 3). In addition, EGF-mediated Akt activation is associated with Bmi-1 stabilization (Figure 4), and the p38 inhibitor, SB203580, also stabilizes Bmi-1 in H_2_O_2_-treated NSCs and *Atm^-/-^* NSCs (Figure 3, 5, and 6). We conclude that in the absence of ATM, chronic oxidative stress results in the activation of p38, leading to inhibition of the Akt-dependent Bmi-1 stabilizing process, thus causing Bmi-1 to degrade more rapidly. It is also possible that p38 downregulates Bmi-1's ability to bind to chromatin, thus Bmi-1 loses its suppressive effects on p21, which is supported by our findings that the p38 inhibitor, SB203580, restores H2A ubiquitination (Figure 6C) and restores p21 levels in *Atm^-/-^* NSCs [6].

Bmi-1 previously has been shown transiently co-localized with the centromeres [24]. Furthermore, Bmi-1−chromatin association has been proven by immunostaining [25]–[26]. However, mechanisms regulating Bmi-1 chromatin association are still unclear. We performed biochemical fractionation of Bmi-1 to see differential chromatin association and found that more Bmi-1 chromatin association is seen in *Atm^+/+^* NSCs than in *Atm^-/-^* NSCs (data not shown). In normal condition, Bmi-1 chromatin association is strictly regulated by its phosphorylation status in the cell cycle [26]–[27]. Therefore, our data might be explained by the fact that *Atm^-/-^* NSCs show abnormal Akt and p38. We have proven that abnormal p38-Akt signaling contributes to defective NSCs proliferation and self-renewal [6]. Another interesting question to be addressed is whether or not Akt/p38-dependent Bmi-1 phosphorylation is critical for its chromatin association.

It is unclear if Bmi-1 is a p38 substrate from our study, but it has been reported that Bmi-1 could be phosphorylated by 3pk (MAPKAP kinase 3), which is a downstream effector of p38 [26]. Phosphorylation of Bmi-1 by 3pk reduces Bmi-1's ability to bind to chromatin, thus reducing its suppressive effect on p21. Another way in which p38 may regulate Bmi-1 levels is via down-regulation of Akt because Bmi-1 restoration by the p38 inhibitor, SB203580, is accompanied by an increase in Akt activation levels under oxidative stress conditions (Figure 5). This result is supported by another report that describes the oxidative stress-induced activation of p38, which attenuates insulin-like growth factor stimulation of Akt [28]. Our studies also show that Bmi-1 is a substrate of Akt, and up-regulation of p-Akt coincides with up-regulation of p-Bmi-1. Until now, no one has shown that Akt can phosphorylate and up-regulate Bmi-1. Therefore, this will be the first time a direct functional link between Akt and Bmi-1 is made. Further study is under way to make their direct links. The connection between the p38/Akt signaling and Bmi-1 stability in A-T is a newly described angle of ATM signaling that may be relevant to clinical aspects of the this disease.

**Figure 7:**
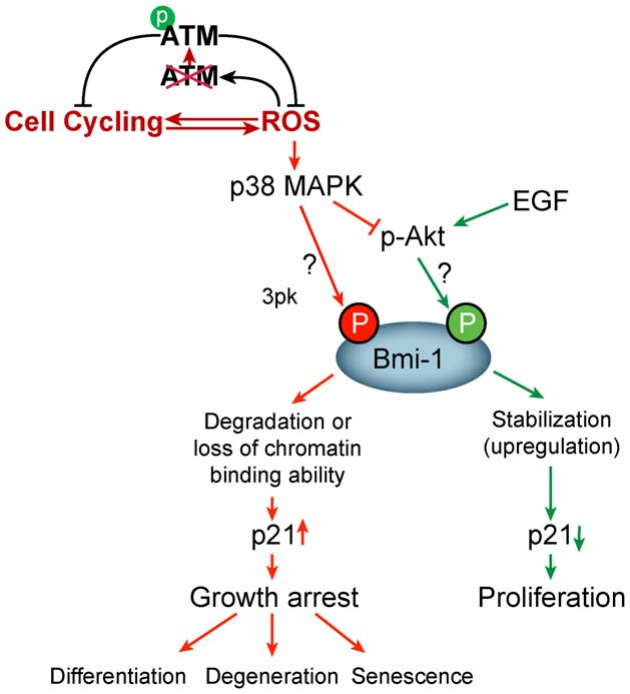
Proposed mechanism underlying defective proliferation of *Atm^-/-^* NSCs. Bmi-1 expression is regulated mainly through posttranscriptional mechanism by proteasomal degradation. EGF-mediated Akt phosphorylation renders Bmi-1 resistant to the degradation process, leading to its stabilization and neural stem cell proliferation (green arrows). However, chronic oxidative stress, in the absence of ATM, activates p38 signaling blocks Akt-dependent Bmi-1 stabilizing, resulting in accelerated proteasome-mediated degradation of Bmi-1 and p21 up-regulation, which in turn may suppress proliferation (red arrow).

Protein kinase activities are redox-sensitive because key cysteine residues in these proteins can undergo posttranslational modifications by oxidants [5]. It has recently been shown that H_2_O_2_ is directly responsible for disulfide bond formation, which results in a conformational alteration and activation of ATM [29]. This suggests that ATM can be activated in response to increased ROS levels to regulate the redox status of the cells, independently of its response to DNA damage [30]. Our findings demonstrate that in the absence of ATM, chronic oxidative stress results in activation of the p38-Bmi-1-p21 pathway in NSCs. Taken together, we propose a possible mechanism regulating Bmi-1 stability by Akt or p38 signaling (Figure 7). We hypothesize that a function of ATM is to act as a redox modulator. As a consequence of increased ROS levels p38 is activated, which may block the Akt-dependent Bmi-1 stabilizing process. This event accelerates proteasome-mediated degradation of Bmi-1, which in turn upregulates p21 and suppresses proliferation (red arrow). During normal condition, EGF-mediated Akt phosphorylation leads to Bmi-1 stabilization and neural stem cell proliferation (green arrows). By controlling p38 or Akt signaling, which has yin and yang effects on Bmi-1 stability, we may be able to control *Atm^-/-^* NSC proliferation as described in the above mechanism.

NSCs possess a range of actions that can be potentially used for therapy, because they are capable of self-renewal and can differentiate into cells of glial and neuronal lineages in the CNS [31]–[32]. Bmi-1 is also important for maintenance of NSC multipotency, which is required for generating neurons [11], [33], and Bmi-1 overexpression increases neurogenic capacity of NSCs [14]. Previous studies demonstrated that normal p38 signaling is essential for NSC differentiation [34] and activated p38 leads to abnormal differentiation in *NPC1* ^-/-^ (The Niemann-Pick type C1) NSCs [35]. We show here that *Atm^-/-^* NSCs have abnormally activated p38 and lower level of Bmi-1 compared to *Atm* ^+/+^ NSCs. We also observed that *Atm^-/-^* NSCs are capable of undergoing multilineage differentiation, but they show different frequencies of differentiated cells when compared to *Atm* ^+/+^ NSCs. (data not shown). Therefore, we hypothesize that abnormal p38–Bmi-1 signaling in *Atm^-/-^* NSCs may affect their neurogenic capacity, which would change the fate of the progenitor cells. Continuing our investigation, it will be still interesting to examine the fate of the progenitor cells to astroglia, oligodendroglia, and neurons by assessing the frequency of multilineage cells from *Atm* ^+/+^ and *Atm^-/-^* NSCs, and to determine whether p38 signaling inhibition affects the neurogenic capacity of *Atm^-/-^* NSCs.

In recent years, NSC transplantation has proven to be a more tractable therapeutic strategy for neurodegenerative disorders than the conventional placement of differentiated neurons, since NSCs can replace many cell types, not just one, and can synergize with other therapies, including drug treatments [31]–[32], [36]. When NSCs are defective, as they are in *Atm^-/-^* mice, transplanting normal *Atm* ^+/+^ NSCs may rescue impaired NSCs by restoring homeostasis to the NSCs themselves, or by stabilizing normal metabolism and redox balance in the supporting cell microenvironment. The imposition of “molecular homeostasis” may be the basis for this NSC rescue. Here we show that *Atm^-/-^* NSCs have abnormally reduced levels of Bmi-1, and we know that Bmi-1 is necessary for improved NSC survival and proliferation. Thus, our study delineating ATM function in NSCs may provide useful insight for development of stem cell-based novel therapies in human patients with A-T or with other genetic deficiencies that lead to neurodegeneration. Transplantation of normal *Atm* ^+/+^ NSCs into *Atm^-/-^* mouse brains may thus promote a return to normal levels of Bmi-1 in the *Atm^-/-^* NSCs. In future studies, we will develop a reliable NSC transplantation system to ask two questions. First, is *Atm^+/+^* NSC transplantation into the cerebella of *Atm^-/-^* mice followed by a functional recovery of *Atm^-/-^* NSC and restore the normal cerebella architecture? Second, can *Atm^+/+^* NSC transplantation alter p38 signaling and gene expression abnormalities in the NSCs of *Atm^-/-^* mice?

## Materials and Methods

### Mice

The *Atm^-/-^* mice were originally generated by Dr. C. Barlow. They were purchased from the Jackson Laboratory (Bar Harbor, ME). We genotyped offspring of *Atm^+/–^* breeding pairs by real-time polymerase chain reaction–based assays of mouse tail DNA. Littermates were used as controls in all experiments. Animal care was in accordance with The University of Texas MD Anderson Cancer Center guidelines for animal experiments.

### NSC isolation and neurosphere culture

Neurospheres were obtained from P1 pup SVZ, and were maintained in culture essentially as reported [37]. The walls of the lateral ventricles were removed and enzymatically dissociated in HBSS buffer (5 mM KCl, 124 mM NaCl, 3.2 mM MgCl_2_, 100 µM CaCl_2_, 26 mM NaHCO_3_, and 10 mM glucose) containing 1 mg/ml trypsin at 37°C for 10 minutes. The tissue was centrifuged at 750 rpm for 5 minutes in soybean trypsin inhibitor. The dispersed cells were then resuspended in HBSS buffer containing 0.7 mg/ml RNase free DNase and enzymatically disaggregated for 5 minutes at room temperature. The dissociated cells were centrifuged, resuspended in Neural Basal Medium (NBM) containing 20 ng/ml EGF, 10 ng/ml FGF, 2 mM glutamine, antibiotics (100 units/ml penicillin and 100 g/ml streptomycin), and 0.125 µg/ml fungizone, and maintained in an atmosphere of 5% CO_2_, at 37°C. After 1–2 days of incubation, the cells formed neurospheres. Subcultures were prepared every 4–5 days by centrifugation of the neurospheres and dissociation of the cells in 1 ml of trypsin; then, the single-cell suspensions were replated in new culture dishes in fresh medium, to obtain new neurospheres. Experiments were performed with cultured cells between passages 2 and 5. GSCs were developed by Dr. F. Lang (MD Anderson Cancer Center) and were kindly provided by Dr. O. Bogler (MD Anderson Cancer Center). GSCs culture condition was same with *Atm* ^+/+^ and *Atm^-/-^* NSCs as described above.

### Chemical reagents

The PI3K inhibitor LY294001 (10 µM), the p38 MAPK inhibitor SB203580 (10 µM), the ERK inhibitor PD98059 (10 µM), MG132 (10 µM), and Okadaic acid (10 µM), were purchased from BD Biosciences. Lambda phosphatase was purchased from New England Biolabs. Transcription inhibitor cycloheximide (50 µg/ml) was purchased from Sigma-Aldrich.

### Generation of Bmi-1 over-expressing *Atm* ^+/+^ and *Atm* ^-/-^ neural stem cells

Bmi-1 bearing retrovirus was made using a mouse stem cell virus (MSCV) vector MSCV-GFP-Bmi-1 (kindly provided by Dr. Morrison, University of Michigan), which contains an internal ribosome entry site (IRES) followed by GFP [14]. Preparation of the virus was carried out by transfection of 293T packaging cells with the MSCV-GFP-Bmi-1 vector. 293T cells were allowed to produce virus for 24–48 hours, then cell supernatants were collected, 0.2 micron filtered, and stored at −80°C or used fresh to infect cultured NSCs. To generate Bmi-1 over-expressing *Atm* ^+/+^ and *Atm^-/-^* NSCs, neonatal subventricular zone (SVZ) tissue from wild type or Atm deficient mice was dissected, dissociated, and cultured as described above. NSCs were plated at 20,000 cells per well of a six-well plate. After 24–48 hours, virus-containing supernatant was added to NSC cultures for 16 hours. The culture medium was then replaced for 24 hours, and cells were trypsinized and re-plated for tests of proliferative capacity. Before proliferative capacity assays, MSCV-GFP-Bmi-1 expression in cells was assessed by fluorescence microscopy.

### Cell proliferation assays

After trypsinizing neurospheres, single neural stem cells were resuspended in NBM containing 20 nM EGF and 20 nM FGF. The cells were then seeded into 96-well plates at a density of 10^2^ cells per well, to allow cell communication by paracrine/autocrine factors for physiological proliferation. After incubation for 48 hours, the numbers of newly formed neurospheres per well were counted, using phase contrast microscopy. To measure neurosphere sizes, images were obtained from 3 separate fields per well, and the volumes of neurospheres estimated using a microimage analysis system from Olympus. To measure total cell proliferation in neurospheres, cells in each well were trypsinized, and total cell numbers per well were counted.

### Immunocytochemical image analysis

Neurospheres were seeded on 2– or 8– well chamber slides coated with poly-L-lysine/lamin, and then incubated for 3 hours, to facilitate adhesion. These cells, which will be referred to as “adhered neurospheres” in the text, maintained the properties of undifferentiated cells, as evidenced by anti-nestin staining. Adhered neurospheres also respond to H_2_O_2_ in a manner similar to the floating neurospheres. For ROS response experiments, neurospheres in monolayer cultures were treated with H_2_O_2_ in NBM for 16 hours, after which the cells were fixed with 4% paraformaldehyde in PBS for 30 minutes at room temperature, and permeabilized with 0.1% Triton X-100 in PBS for 15 minutes. For staining with antibodies, the cells were preincubated for 2 hours at 37°C with 10% FBS in PBS, and then further incubated at 4°C overnight with primary antibodies. The antibody-bound cells were washed three times in PBS for 5 minutes each, and fluorescent secondary antibodies were added for 1 hour at 37°C. Nuclei were stained with 4′, 6-diamidino-2-phenylindole (DAPI). The stained slides were then mounted in Slowfade (Molecular Probes). Cells were imaged under an Olympus IX2-SL microscope, equipped with a ×400 objective and an Olympus DP 70 digital camera connected to a PC computer.

### Protein analysis

For protein measurement, cells were seeded into 10-cm dishes, and treated with drugs or H_2_O_2_. At indicated times after treatment, cells were collected and washed once with ice-cold PBS and then resuspended into lysis buffer containing 150 mM NaCl, 0.5% w/v sodium dodecyl sulfate (SDS), 0.5% v/v NP-40, 0.5% w/v sodium deoxycholate, 1 mM EGTA, and a mixture of protease inhibitors (Complete Mini tablets; Boehringer Mannheim). For Western blotting, protein concentrations were determined using a Bradford reagent (BioRad). Proteins (30 µg) were separated by SDS-polyacrylamide gel electrophoresis on 10% gels, and transferred to polyvinylidene difluoride membranes prior to incubation with primary antibodies.

### Antibodies

Antibodies used for Western blotting analysis were anti-phospho-Akt (Ser473), anti-Akt, anti-phospho-p38 (Th180/Tyr182), anti-p38, anti-ubiquitinated histone H2A, anti-Akt substrate (Cell Signaling Technology); anti-p21 and anti-β-actin (Santa Cruz Biotechnology); anti-Bmi-1 (Millipore Co.).

### Quantitative RT-PCR

Expression levels for *bmi-1*, *p21^Cip1^*, *p16^Ink4a^*, and *p19^Arf^* were quantified, relative to *gapdh*, internal RNA control, by quantitative RT-PCR. Sequences of PCR primers are listed as follows. Primers that amplified *bmi-1*: Mm03053308_g1. Primers that amplified *p21Cip1*: cdkn1a (p21): Mm00432448_m1. Primers that amplified *p16Ink4a:* Mm01257348_m1. Primers that amplified *p19Arf* were designed: sense, 5′-GGGCCGCACCGGAAT-3′ and antisense, 5′-AAGAGCTGCTACGTGAACGT-3′.

### Statistics

For each experiment, data are presented as the mean ± S.E. of values. Each experiment was repeated at least three times. Statistical comparisons of values for *Atm* ^+/+^ *vs*. *Atm^-/-^* neurospheres, and for untreated control *vs*. treated neurospheres were made using an analysis of variance (ANOVA), followed by Bonferroni's *post hoc* test. Differences were considered significant when *p*<0.05. Analyses of data were performed using Prism 5 Software (GraphPad Software, Inc. San Diego, CA).

### Differentiation of neural stem cells

Neurospheres were enzymatically dissociated as described above. The cells were seeded on to chamber slides, and then maintained in medium containing 10% FBS, without EGF and FGF for 7 days. Antibodies used for characterization of neural stem cells were anti-Map2 (Cell Signaling Technology), anti-GFAP (Santa Cruz Biotechnology).

## Supporting Information

Figure S1 **Bmi-1 downregulated in** ***Atm^-/-^*** **NSCs** *Atm* ^+/+^ and *Atm^-/-^* neurospheres were analyzed for phospho-p38, phospho-Akt, Bmi-1, and p21 by Immunofluorescence. Cells were counterstained by DAPI (4′-6-Diamidino-2-phenylindole), which identifies the nuclei of the NSCs.
(DOC)

## References

[1] Allen DM, van Praag H, Ray J, Weaver Z, Winrow CJ (2000). Ataxia telangiectasia mutated is essential during adult neurogenesis.. Genes Dev.

[2] Gage FH (2000). Mammalian neural stem cells.. Science.

[3] Chen P, Peng C, Luff J, Spring K, Watters D (2003). Oxidative stress is responsible for deficient survival and dendritogenesis in purkinje neurons from ataxia-telangiectasia mutated mutant mice.. J Neurosci.

[4] Liu N, Stoica G, Yan M, Scofield VL, Qiang W (2005). ATM deficiency induces oxidative stress and endoplasmic reticulum stress in astrocytes.. Lab Invest.

[5] Kim J, Wong PK (2009). Oxidative Stress Is Linked to ERK1/2-p16 Signaling-mediated Growth Defect in ATM-deficient Astrocytes.. J Biol Chem.

[6] Kim J, Wong PK (2009). Loss of ATM Impairs Proliferation of Neural Stem Cells Through Oxidative Stress-Mediated p38 MAPK Signaling.. Stem Cells.

[7] Molofsky AV, Pardal R, Iwashita T, Park IK, Clarke MF (2003). Bmi-1 dependence distinguishes neural stem cell self-renewal from progenitor proliferation.. Nature.

[8] Molofsky AV, He S, Bydon M, Morrison SJ, Pardal R (2005). Bmi-1 promotes neural stem cell self-renewal and neural development but not mouse growth and survival by repressing the p16Ink4a and p19Arf senescence pathways.. Genes Dev.

[9] Li Z, Cao R, Wang M, Myers MP, Zhang Y (2006). Structure of a Bmi-1-Ring1B polycomb group ubiquitin ligase complex.. J Biol Chem.

[10] Leung C, Lingbeek M, Shakhova O, Liu J, Tanger E (2004). Bmi1 is essential for cerebellar development and is overexpressed in human medulloblastomas.. Nature.

[11] Fasano CA, Dimos JT, Ivanova NB, Lowry N, Lemischka IR (2007). shRNA knockdown of Bmi-1 reveals a critical role for p21-Rb pathway in NSC self-renewal during development.. Cell Stem Cell.

[12] Liu J, Cao L, Chen J, Song S, Lee IH (2009). Bmi1 regulates mitochondrial function and the DNA damage response pathway.. Nature.

[13] Chatoo W, Abdouh M, David J, Champagne MP, Ferreira J (2009). The polycomb group gene Bmi1 regulates antioxidant defenses in neurons by repressing p53 pro-oxidant activity.. J Neurosci.

[14] He S, Iwashita T, Buchstaller J, Molofsky AV, Thomas D (2009). Bmi-1 over-expression in neural stem/progenitor cells increases proliferation and neurogenesis in culture but has little effect on these functions in vivo.. Dev Biol.

[15] Bhattacharyya J, Mihara K, Yasunaga S, Tanaka H, Hoshi M (2009). BMI-1 expression is enhanced through transcriptional and posttranscriptional regulation during the progression of chronic myeloid leukemia.. Ann Hematol.

[16] Chen W, Sun Z, Wang XJ, Jiang T, Huang Z (2009). Direct interaction between Nrf2 and p21(Cip1/WAF1) upregulates the Nrf2-mediated antioxidant response.. Mol Cell.

[17] Torroglosa A, Murillo-Carretero M, Romero-Grimaldi C, Matarredona ER, Campos-Caro A (2007). Nitric oxide decreases subventricular zone stem cell proliferation by inhibition of epidermal growth factor receptor and phosphoinositide-3-kinase/Akt pathway.. Stem Cells.

[18] Stock JK, Giadrossi S, Casanova M, Brookes E, Vidal M (2007). Ring1-mediated ubiquitination of H2A restrains poised RNA polymerase II at bivalent genes in mouse ES cells.. Nat Cell Biol.

[19] Li Z, Cao R, Wang M, Myers MP, Zhang Y (2006). Structure of a Bmi-1-Ring1B polycomb group ubiquitin ligase complex.. J Biol Chem.

[20] Zuluaga S, Alvarez-Barrientos A, Gutiérrez-Uzquiza A, Benito M, Nebreda AR (2007). Negative regulation of Akt activity by p38alpha MAP kinase in cardiomyocytes involves membrane localization of PP2A through interaction with caveolin-1.. Cell Signal.

[21] Westermarck J, Li SP, Kallunki T, Han J, Kähäri VM (2001). p38 mitogen-activated protein kinase-dependent activation of protein phosphatases 1 and 2A inhibits MEK1 and MEK2 activity and collagenase 1 (MMP-1) gene expression.. Mol Cell Biol.

[22] Gonzalez I, Tripathi G, Carter EJ, Cobb LJ, Salih DAM (2004). Akt2, a Novel Functional Link between p38 Mitogen-Activated Protein Kinase and Phosphatidylinositol 3-Kinase Pathways in Myogenesis.. Mol Cell Biol.

[23] Lee B, Kim CH, Moon SK (2006). Honokiol causes the p21WAF1-mediated G(1)-phase arrest of the cell cycle through inducing p38 mitogen activated protein kinase in vascular smooth muscle cells.. FEBS Lett.

[24] Soulez M, Saurin AJ, Freemont PS, Knight JC (1999). SSX and the synovial-sarcoma specific chimaeric protein SYT-SSX co-localize with the human Polycomb group complex.. Oncogene.

[25] Obuse C, Yang H, Nozaki N, Goto S, Okazaki T (2004). Proteomics analysis of the centromere complex from HeLa interphase cells: UV-damaged DNA binding protein 1 (DDB-1) is a component of the CEN-complex, while BMI-1 is transiently co-localized with the centromeric region in interphase.. Genes Cells.

[26] Voncken JW, Niessen H, Neufeld B, Rennefahrt U, Dahlmans V (2005). MAPKAP kinase 3pK phosphorylates and regulates chromatin association of the polycomb group protein Bmi1.. J Biol Chem.

[27] Voncken JW, Schweizer D, Aagaard L, Sattler L, Jantsch MF (1999). Chromatin-association of the Polycomb group protein BMI1 is cell cycle-regulated and correlates with its phosphorylation status.. J Cell Sci.

[28] Davila D, Torres-Aleman I (2008). Neuronal death by oxidative stress involves activation of FOXO3 through a two-arm pathway that activates stress kinases and attenuates insulin-like growth factor I signaling.. Mol Biol Cell.

[29] Guo Z, Kozlov S, Lavin MF, Person MD, Paull TT (2010). ATM activation by oxidative stress.. Science.

[30] Wong PK, Kim J, Kim SJ, Kuang X, Lynn WS, McNeil AS (2010). Oxidative stress-mediated neurodegeneration: A tale of two models.. Neurodegeneration: Theory, disorders and treatments.

[31] Parker MA, Anderson JK, Corliss DA, Abraria VE, Sidman RL (2005). Expression profile of an operationally-defined neural stem cell clone.. Exp Neurol.

[32] Lee JP, Jeyakuma M, Gonzalez R, Takahashi H, Lee PJ (2007). Stem cells act through multiple mechanisms to benefit mice with neurodegenerative metabolic disease.. Nat Med.

[33] Fasano CA, Phoenix TN, Kokovay E, Lowry N, Elkabetz Y (2009). Bmi-1 cooperates with Foxg1 to maintain neural stem cell self-renewal in the forebrain.. Genes Dev.

[34] Androutsellis-Theotokis A, Leker RR, Soldner F, Hoeppner DJ, Ravin R (2006). Notch signaling regulates stem cell numbers in vitro and in vivo.. Nature.

[35] Yang SR, Kim SJ, Byun KH, Hutchinson B, Lee BH (2006). NPC1 gene deficiency leads to lack of neural stem cell self-renewal and abnormal differentiation through activation of p38 mitogen-activated protein kinase signaling.. Stem Cells.

[36] Li J, Imitola J, Snyder EY, Sidman RL (2006). Neural stem cells rescue nervous purkinje neurons by restoring molecular homeostasis of tissue plasminogen activator and downstream targets.. J Neurosci.

[37] Molofsky AV, Slutsky SG, Joseph NM, He S, Pardal R (2006). Increasing p16INK4a expression decreases forebrain progenitors and neurogenesis during ageing.. Nature.

