# Supplementary material for: p38 MAPK-Mediated Bmi-1 Down-Regulation and Defective Proliferation in ATM-Deficient Neural Stem Cells Can Be Restored by Akt Activation
Source: PLoS One. 2011 Jan 28;6(1):e16615. doi: 10.1371/journal.pone.0016615 (PMC3030607; doi:10.1371/journal.pone.0016615)

**Figure S 1. Bmi-1 downregulated in *Atm^-/-^*NSCs**

*Atm*^+/+^ and *Atm^-/-^*neurospheres were analyzed for phospho-p38, phospho-Akt, Bmi-1, and p21 by Immunofluorescence. Cells were counterstained by DAPI (4'-6-Diamidino-2-phenylindole), which identifies the nuclei of the NSCs.


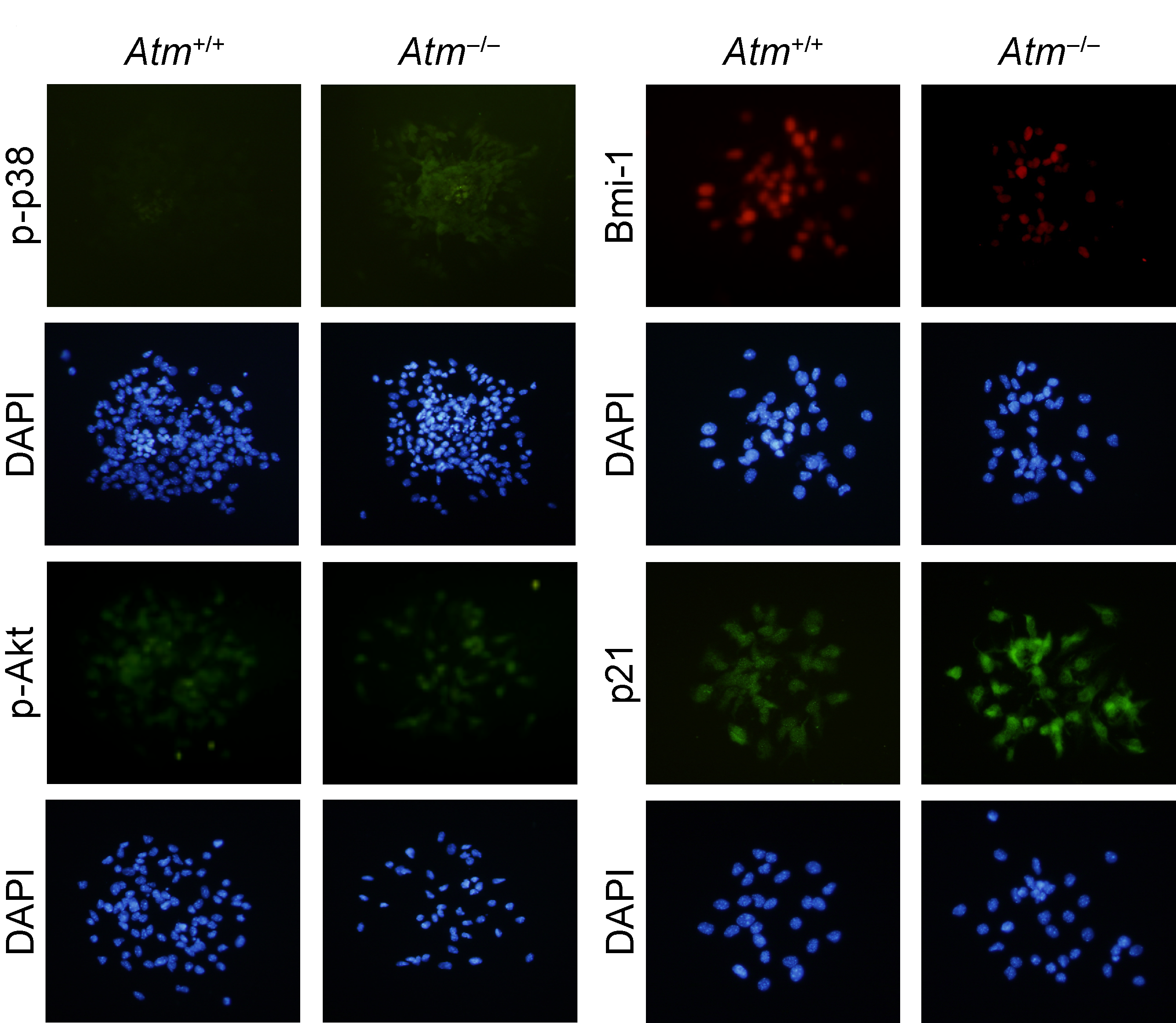

Supplement: Figure S1 — Bmi-1 downregulated in Atm-/- NSCs Atm +/+ and Atm-/- neurospheres were analyzed for phospho-p38, phospho-Akt, Bmi-1, and p21 by Immunofluorescence. Cells were counterstained by DAPI (4′-6-Diamidino-2-phenylindole), which identifies the nuclei of the NSCs. (DOC) [file pone.0016615.s001.doc]
